# Cryo-EM structure of *Mycobacterium smegmatis* ribosome reveals two unidentified ribosomal proteins close to the functional centers

**DOI:** 10.1007/s13238-017-0456-9

**Published:** 2017-09-05

**Authors:** Zhifei Li, Xueliang Ge, Yixiao Zhang, Lvqin Zheng, Suparna Sanyal, Ning Gao

**Affiliations:** 1 State Key Laboratory of Membrane Biology, Peking-Tsinghua Joint Center for Life Sciences, School of Life Sciences, Peking University, Beijing, 100871 China; 2 Beijing Advanced Innovation Center for Structural Biology, Tsinghua-Peking Joint Center for Life Sciences, School of Life Sciences, Tsinghua University, Beijing, 100084 China; 3 Department of Cell and Molecular Biology, Uppsala University, BMC, Box-596, Uppsala, Sweden

## Dear Editor,

*Mycobacterium smegmatis* is commonly used as a laboratory surrogate in studying the physiology and pathogenesis of disease-causing mycobacteria, including *Mycobacterium tuberculosis*, which causes tuberculosis (TB) in human. Nearly half of the existing antibiotics target cellular protein biosynthesis to kill or inhibit the growth of bacteria (Wilson, 2014). Owing to its great potential as a therapeutic target for antimicrobial drugs, the structures of the ribosomes from pathogenic microbes and model organisms have been of major interest for several decades [for reviews, see (Ramakrishnan, 2014; Yusupova and Yusupov, 2017)]. So far, only low-resolution cryo-EM structures were available for the mycobacterial ribosomes; *M*. *smegmatis* 70S, 12 Å (FSC = 0.5 criterion) (Shasmal and Sengupta, 2012) and the *M*. *tuberculosis* 50S, 8.5 Å (FSC = 0.143 criterion) (Li et al., 2015). These structures have revealed a few interesting differences from other bacterial ribosomes, particularly the length variation of certain rRNA helices (Shasmal and Sengupta, 2012; Li et al., 2015). However, they are not in sufficient resolution to depict the essential structural differences that might contribute to the designing of the mycobacterial ribosome specific anti-TB drugs.

To gain atomic details of mycobacterial ribosomes, we first purified the 70S ribosome from *M*. *smegmatis* (mc^2^155 strain) (*MS*70S) (Fig. S1). An *in vitro* reconstituted translation system based on *M*. *smegmatis* components was developed to check the activity of the ribosomes in peptide bond formation and tRNA translocation. The activity of the purified *MS*70S, determined from the dipeptide (fMet-Leu) synthesis assay, was 50% (Fig. S2A), likely due to the fact the buffer was optimized for *E*. *coli* translation assays. The *MS*70S ribosomes also formed tripeptide (fMet-Leu-Leu) with *MS* EF-G, with a rate *k*_*obs*_ = 1.36 ± 0.12 s^−1^, comparable to *E*. *coli* ribosomes under similar conditions (*k*_*obs*_ = 2.8 ± 0.2 s^−1^) (Fig. S2B). Next, cryo-EM single-particle analysis was employed to determine its structure. After a cascade of 2D and 3D classification (Fig. S3), we were able to obtain a near atomic structure for the *MS*70S. Mask-based refinement resulted in two improved density maps for the *MS*30S and *MS*50S at the resolution of 3.45 Å and 3.08 Å, respectively (Fig. S4). Most rRNA residues and protein side chains in the *MS*50S and *MS*30S are clearly separated (Fig. S5).

**Figure 1:**
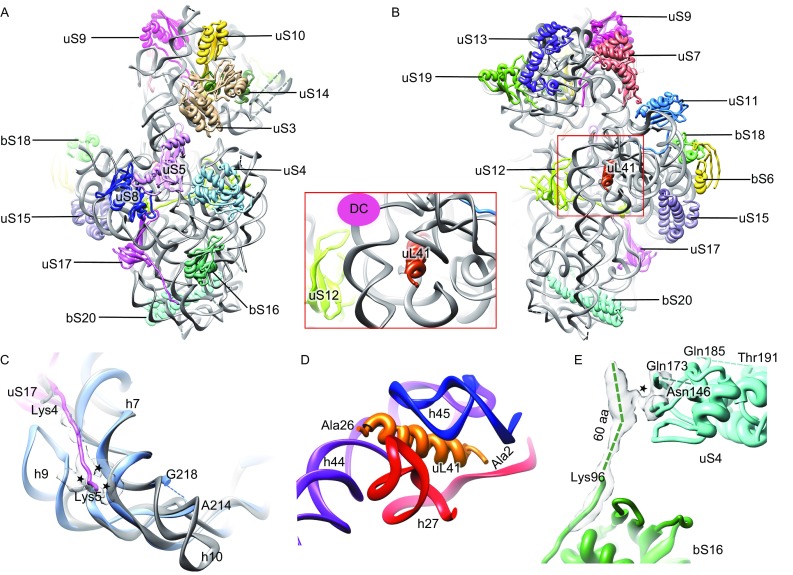
**Overall atomic model and unique features of the** ***MS*** **30S**. (A and B) Atomic model of the *MS*30S, viewed from the solvent surface (A) and intersubunit surface (B). Inset, zoom-in view of uL41 close to the decoding center (DC). (C) The extended N-terminus of uS17 interacts with h7 and h9. Counterparts in the *EC*70S are colored gray. The coordinates of the *E*. *coli* components are from a crystallography study (PDB 4KIY) (Pulk and Cate, 2013). (D) Ribosomal protein uL41 is surrounded by h27, h44, and h45. (E) The extended C-terminus of bS16 interacts with globular domain of uS4. The interaction sites are indicated by asterisks

With the *MS*30S map, we modeled 19 proteins and more than 90% of 16S rRNA residues (Fig. 1A, 1B and Table S1). Compared to the *E*. *coli* 70S ribosome (*EC*70S), h10 and h17 are distinctively shorter in the *MS*70S (Figs. 1C and S6C), while h9 is about 17-nt longer (Figs. 1C and S7B). Notably, there is no density present at the expected position of bS21 in the 30S map and no bS21 sequence could be found in the *M*. *smegmatis* protein database. In addition, we did not observe strong density for bS1 in the *MS*70S as well. Protein bS1 is a flexible component of the 30S subunit at the mRNA exit site and plays a pivot role in translation initiation on canonical mRNAs by interacting with the 5′-UTR to facilitate unfolding of secondary structures (Byrgazov et al., 2015). The absence of bS21 and weak association of bS1 in the *MS*70S seem to correlate with the fact that non-canonical translation with leaderless mRNA (without 5′-UTR and Shine Dalgarno sequence) contributes to a distinctly large portion (~25%) of mycobacterial proteome (Shell et al., 2015).

Interestingly, a rod-like density, which is absent from other known prokaryotic ribosomes such as the *EC*70S (Pulk and Cate, 2013), *T*. *thermophilus* 70S (*TT*70S) (Selmer et al., 2006), and *Staphylococcus aureus* 70S (*SA*70S) (Khusainov et al., 2016), locates in a pocket formed by h27, h44, and h45 close to the decoding center (DC) in the 30S subunit (Figs. 1B, 1D and S5E). This position is reminiscent of protein eL41 in eukaryotic ribosomes, which creates a central inter-subunit bridge (Ben-Shem et al., 2011). A survey of mycobacterial protein database indeed located the identity and sequence of this unannotated protein (UniProt # A0QR10 for *M*. *smegmatis* and UniProt # P9WKT5 for *M. tuberculosis*). The flexible C-terminus points to the 50S subunit. We propose to change the name of protein eL41 to uL41, because it is no longer a eukaryote-specific ribosomal protein. The close contact of uL41 with decoding helices (Fig. 1D), such as h44 and h27 (Fig. S8), may have a profound effect on mycobacterial translation, particularly in the decoding steps.

Almost all of the 30S proteins we have modeled have conserved globular domain, and more than half of them have variable N- or C-terminus compared with other bacterial species (Table S1). Particularly, many of them have extended terminal sequences in *M*. *smegmatis*, and these extensions are even longer in *M*. *tuberculosis*. For example, protein uS17 bears an extended N-terminus (~13 residues) (Fig. S6A), which establishes a species-specific interaction with h7 and h9 of the 16S rRNA (Fig. 1C). Notably, this N-terminus is even longer in *M*. *tuberculosis* (Fig. S6A). Another example is the mycobacterial bS16 protein, which possesses a very long C-terminus (about 60 residues longer than *E*. *coli* bS16). The extension is not resolved in atomic resolution, but apparently it forms species-specific interaction with the globular domain of uS4 (Figs. 1E and S6B). Besides 30S body proteins (bS16 and uS17), a few proteins in the neck region of the 30S have extended N- and/or C-terminus, including uS3 and uS5 (at the mRNA entry site), and uS11 (at the mRNA exit site) (Table S1). As these N- and C-termini are quite flexible, we were not able to build any of them (Table S1). Nevertheless, one of them, the N-terminal extension of uS5 is expected to be in the proximity of the mRNA entry site. Altogether, the compositional difference of the *MS*30S and unique extensions of mycobacterial ribosomal proteins might reflect the physiological difference of mycobacteria with regard to their widespread non-canonical translation on leaderless mRNA (Cortes et al., 2013; Shell et al., 2015).

**Figure 2:**
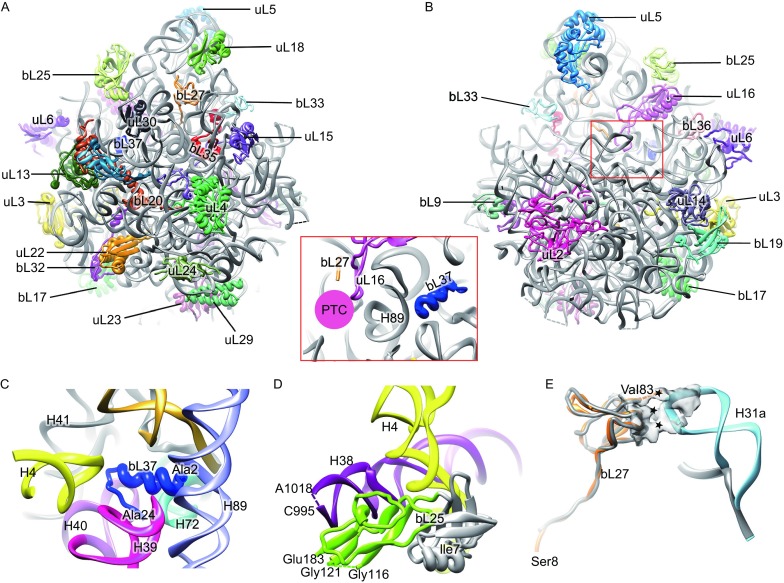
**Overall atomic model and unique features in the** ***MS*** **50S**. (A and B) Atomic model of the *MS*50S, viewed from solvent surface (A) and inter-subunit surface (B). Inset, relative orientation of bL27 with respect to the peptidyl transferase center (PTC). (C) Ribosomal protein bL37 is encircled by H4, H39, H40, base of H41 and H42, H72 and H89. (D) The extra C-terminal domain of bL25 is in proximity to H38 (purple). Conserved N-terminal domain of bL25 is colored gray while the extra C-terminal domain is colored green. (E) The extended H31 interacts with bL27, N-terminus of which extends to the peptidyl transfer center. The interaction sites are indicated by asterisks. Protein bL27 and H31a in the *MS*70S are colored with orange and cyan, respectively, while their counterparts in the *EC*70S are in gray. The coordinates of the *E*. *coli* components are from a crystallography study (PDB 4KIX) (Pulk and Cate, 2013)

For the *MS*50S, we modeled 29 proteins and about 84% of 23S rRNA residues (Fig. 2A, 2B and Table S2). An unexpected finding is the presence of a piece of additional density in the 50S map. After rounds of “trial and error” attempts with small ORFs in the *M*. *Smegmatis* databse, a protein of 24 amino acids (UniProt # A0QTP4) was found to match very well with the density (Figs. S5A and S9). This protein, consisting of an α-helical N-terminus (~13 residues) and a C-terminal loop, is encircled by a pocket formed by H39, H40, joint of H41 and H42, H72 and H89 (Fig. 2C), while in other prokaryotic ribosomes (*EC*70S, *TT*70S, and *SA*70S), there is no protein in this pocket. Located deep inside an rRNA pocket, it is a previously unidentified and uncharacterized ribosomal protein; we propose to name it as bL37. Bioinformatic analysis shows that this bL37 only exists in high G + C, actinobacterial species, including mycobacteria (Fig. S10B and S10C). Evolutionary relationship based 16S-like rRNA sequences confirms that bL37-containing species are in a same bacterial branch that is separated from a few well-studied species, such as *E*. *coli*, *T*. *thermophilus* and *B*. *subtilis* (Fig. S11). Notably, bL37 strongly interacts with the buried side of a helix H89 at the peptidyl transferase center (Figs. 2, inset and S7C). Therefore, bL37 might be able to affect the peptidyl transferase function of the 50S subunit, likely through modulating H89 conformation.

Among all modelled 50S proteins, about 40% of them have variable N- or C-terminus in the *MS*50S (Table S2). For example, protein bL25 of *M*. *smegmatis*, similar as those of *Bacillus subtilis*, *T*. *thermophilus* and *S*. *aureus*, has two domains while it only has one domain in *E*. *coli.* The extra C-terminal domain of mycobacterial bL25, locates near H38 in the *MS*50S (Fig. 2D). This domain is involved in tRNA proofreading in *T*. *thermophilus* (Jenner et al., 2010). While in *B*. *subtilis*, bL25 is only expressed and bind to ribosome under stress conditions (Schmalisch et al., 2002).

As to the 23S rRNA of the *MS*50S, some variable helices from the solvent surface, mainly clustered in the L1 stalk region, are remarkably extended. Consistent with previous low-resolution structure (Shasmal and Sengupta, 2012), the most distinct one is H54a. This helix stretches all the way from the bottom of the L1 stalk base to a region near bS6 in the *MS*30S (open state) (Fig. S7) (Shasmal and Sengupta, 2012). Strikingly, in the structure of the *M*. *tuberculosis* 50S, this helix is pointing to a different direction: it locates on the inter-subunit side of the 50S subunit, with its tip occupying the exit site of tRNA (E-site) on the 50S subunit (close state) (Li et al., 2015). Thus, this helix has to be in the open state before the 50S subunit can bind to the 30S subunit, suggesting the presence of additional steps on translation initiation by regulating the structural dynamics of H54a in mycobacteria.

In addition, two other helices H15 and H16a, which do not exist in the *EC*70S, are located close to the base of the L1 stalk (Fig. S7). However, both of them are rather flexible and could not be modeled at the residue precision. Nevertheless, their proximity to the L1 stalk implies that these extended helices might be involved in regulating the dynamics of the L1 stalk during translation cycle. Another lengthened component is H31a (compared to the *EC*70S, *SA*70S, *TT*70S), which creates a set of unique interactions with protein bL27 (Figs. S7 and 2E). Since the N-terminus of bL27 inserts into the peptidyl transfer center, the addition interactions between bL27 and H31a might affect certain kinetic steps of translation elongation on the mycobacterial ribosome.

In summary, we report the near atomic structure of the ribosome from genus mycobacterium, many of which (*M*. *tuberculosis*, *M*. *leprae*, *M*. *avium*) are high-risk pathogens for human. In particular, the unique H54a may play a role in regulating subunit association. Similarly, the two newly discovered proteins uL41 and bL37, located close to the DC and PTC respectively, may have implications in modulating the main functions of the ribosome. The structure provides a resource for studying mycobacterial translation as well as in designing anti-TB drugs.

## FOOTNOTES

While this work was in the stage of manuscript submission, a similar structure was published (Hentschel et al., 2017). The major conclusions related to the structure are highly consistent to our present work.

The cryo-EM density map of the *MS*30S and *MS*50S has been deposited in the EMDB with accession number EMD-6790 and EMD-6789. The atomic model has been deposited in the PDB with accession number 5XYU and 5XYM.

We thank the Tsinghua University Cryo-EM Facility of China National Center for Protein Sciences (Beijing) for providing resources for data collection and computation. Part of the computation was performed on the Computing Platform of the Center for Life Science, Peking University. This work was supported by the National Natural Science Foundation of China (Grant Nos. 31630087, 31422016 and 31470722 to N.G.); the Swedish Research Council (Diary No. 2013-8778, 2014-4423, 2016-06264 and 2008-6593), and the Knut and Alice Wallenberg Foundation (KAW 2011.0081) to S.S.

Zhifei Li, Xueliang Ge, Yixiao Zhang, Lvqin Zheng, Suparna Sanyal, and Ning Gao declare that they have no conflict of interest. This article does not contain any studies with human or animal subjects performed by the any of the authors.

## Electronic supplementary material

Below is the link to the electronic supplementary material.

Supplementary material 1 (PDF 6472 kb)
