# Supplementary material for: Cryo-EM structure of Mycobacterium smegmatis ribosome reveals two unidentified ribosomal proteins close to the functional centers
Source: Protein Cell. 2017 Sep 5;9(4):384–8. doi: 10.1007/s13238-017-0456-9 (PMC5876184; doi:10.1007/s13238-017-0456-9)
Supplement: Supplementary file 1 — Supplementary material 1 (PDF 6472 kb) [file 13238_2017_456_MOESM1_ESM.pdf]

Methods

Ribosome purification

M. smegmatis mc^{2}155 strain was grown at 37°C in Middlebrook 7H9 liquid medium supplemented with 0.2% glycerol, 0.05% Tween 80 and harvested in the late exponential phase at an $OD_{600\ nm}$ = 0.8. *M. smegmatis* cells pellets (40 g) were resuspended in 140 mL lysis buffer (20 mM Tris-HCl pH 7.6, 60 mM NH_4Cl, 10.5 mM magnesium acetate, 0.5 mM EDTA, 3 mM β-mercaptoethanol and 0.5 µg/ml DNase I) and broken by Ultra-sonication followed by French press. The cell lysate was centrifuged for 2 × 40 min at 16,000 rpm in a Fiberlite™ FS21-8×50 rotor. The supernatant was subjected to ultracentrifugation on a sucrose cushion (1.1 M sucrose, 20 mM Tris-HCl pH 7.6, 500 mM NH_4Cl, 10.5 mM magnesium acetate, 0.5 mM EDTA, 3 mM β-mercaptoethanol) spinning for 19 hours at 28,000 rpm in a Beckman coulter Ti 50.2 rotor. The crude ribosome pellets were resuspended in wash buffer (20 mM Tris-HCl pH 7.6, 500 mM NH_4Cl, 10.5 mM magnesium acetate, 0.5 mM EDTA, 7 mM β-mercaptoethanol) and then applied to a second sucrose cushion centrifugation for 19 hours at 28,000 rpm. Ribosome pellets were then dissolved in overlay buffer (20 mM Tris-HCl pH 7.6, 60 mM NH_4Cl, 5.25 mM magnesium acetate, 0.25 mM EDTA, 3 mM β-mercaptoethanol), subsequently separated by zonal centrifugation in 10% to 40% m/v sucrose gradient in Beckman coulter Ti 15 rotor for 16 hours at 26,000 rpm in the overlay buffer. The 70S fractions (Figure S1A) were collected and confirmed by agarose gel electrophoreses (Figure S1B). The ribosomes were further pelleted in Beckman coulter Ti 50.2 rotor by centrifugation for 24 hours at 35,000 rpm. The pure 70S ribosome was dissolved in HEPES - polymix buffer (Mandava et al., 2012) and stored in -80°C.

Biochemical assays for ribosomal activity in di and tripeptide formation

The translation factors (IF1, IF2, IF3, EF-Tu, EF-Ts and EF-G) were cloned from the genomic DNA of *M. smegmatis* mc^{2}155 strain in pET24a expression vector with CTD $(His)_6$-tag. The proteins were over-expressed in *E. coli* BL21-CodonPlus (DE3)-RIPL and affinity purified with His-trap column followed by gel filtration with Sephadex G75 column. The [^{3}H]-fMet-$tRNA^{fMet}$, $tRNA^{Leu}$ and mRNA coding for Met-Leu-Leu-Stop were prepared as described earlier (Degiacomi et al., 2016; Holm et al., 2016). For single round di- and tri-peptide formation, the reactions were performed in HEPES-polymix buffer with additions of 10 mM phosphoenol pyruvate, 1mM ATP and GTP, 50 µg/ml pyruvate kinase and 2 µg/ml myokinase for energy regeneration. For checking ribosomal activity in dipeptide formation, an initiation complex (IC) was formed with 70S ribosome (0.5 - 5 µM), 2 µM IFs, 10 µM mRNA and 1 µM [^{3}H]-fMet-$tRNA^{fMet}$ by incubating at 37 °C for 15 min. In parallel, an elongation mix (EM) was prepared, by incubating 50 µM EF-Tu, 20 µM EF-Ts, 20µM $tRNA^{Leu}$, 0.5 mM Leu and 1 unit Leu-tRNA synthetase at 37°C for 15 min. An equal volume of

the IC and EM was mixed at 37°C for 10 s and quenched with 17% formic acid. The dipeptides were isolated and analysed in HPLC as described earlier (Degiacomi et al., 2016; Holm et al., 2016). The activity of the ribosome was determined by fitting the data with hyperbolic function and estimating the reciprocal of the minimal ribosome concentration needed to reach the saturation (Figure S2A). For tri-peptide formation, an IC with 1 µM 70S ribosome, and an EM with 5 µM EF-G were prepared with all *M. smegmatis* as well as *E. coli* components. After 15-min incubation at 37 °C, equal volumes of IC and EM were rapidly mixed and quenched at different time points using a quench-flow instrument (BioLogic QFM-400). The peptides were analysed as mentioned in (Degiacomi et al., 2016; Holm et al., 2016) . The time curves were fitted with single exponential and the rates were determined.

Sample preparation and data collection

3.5-µL aliquots of purified ribosomes at a concentration of ~100 nM were applied to carbon-coated grids (Quantifoil, R2/2). Grids were blotted with filter paper at both sides for 2.5 s using FEI vitrobot Mark IV (100% humidity, 4°C) and vitrified in liquid ethane. The cryo-grids were transferred to a transmission electron microscope (FEI Titan Krios) operating at 300 kV for data collection. Images were recorded, at a nominal magnification of 22500 X, on Gatan K2 Summit detector in counting mode. Under these conditions, the pixel size at the object scale is 1.32 Å. The nominal defocus used to collect data ranged from -1.5 µm to -2.2 µm. All image stacks were collected with UCSF Image4 (Li et al., 2015) at low-dose conditions with each stack containing 32 dose-fractionated frames. The total exposure time for each stack was 8 s and the dose rate for each pixel was ~8 counts.

Image processing

MOTIONCORR (Li et al., 2013) was used to align the 32-frame stacks and to generate motion-corrected micrographs. The contrast transfer function (CTF) parameters of each micrographs was estimated using CTFFIND3 (Mindell and Grigorieff, 2003). The micrographs were first screened using SPIDER (Shaikh et al., 2008). Particles were picked automatically using SPIDER, and 2D classification was performed with RELION (Scheres, 2012). Particles in good 2D classes (Figure S3A) were chosen for further 3D classification using RELION. To improve the resolution of the density map to facilitate the modeling, several rounds of 3D classification were performed (Figure S2B). In the first round, the differences among the 8 classes were dominated by conformational variations of the 30S subunit (largely inter-subunit rotation) and the presence/absence of tRNA. Six similar classes were combined and used for the second round 3D classification (into three classes). Two of the final three classes were combined for high-resolution 3D refinement. After refinement the density of the 30S subunit was not as good as the 50S subunit, indicating

that the 30S subunit was still heterogeneous in conformation. Then, another round of 3D classification was performed, with a 30S soft-mask applied during classification (Figure S3B). Only one class were selected for the high-resolution refinement for the 30S subunit (47,338 particles). Final rounds of refinement for the 50S and 30S subunits were done with respective soft-masks applied, and with a final set of particles which were re-extracted from the dose-reduced micrographs (frame 2 to 15 using MOTIONCORR). The final resolution estimations for the *MS*50S and MS30S are 3.08 Å and 3.45 Å, respectively (based on the gold-standard FSC = 0.143 criterion). Local resolution variations were estimated using ResMap (Kucukelbir et al., 2014).

Model building and refinement

The modelling was similarly performed as previously described (Wu et al., 2016). The crystal structure of *E. coli* 50S and 30S subunits (PDB accession number 4KIX, 4KIY) (Pulk and Cate, 2013) were fitted in the *MS*50S and *MS*30S maps, respectively. All ribosomal proteins and rRNAs were aligned with their *E. coli* counterparts using Clustal Omega (Sievers et al., 2011) to analyze the conserved regions. For the conserved domains of ribosomal proteins, we mutated the *E. coli* residues to the corresponding *M. smegmatis* residues manually during the model building. The mycobacterium-specific extensions/domains of ribosomal proteins were built *de novo* using COOT (Emsley et al., 2010). For rRNA models, base-pair information was reviewed manually.

The atomic models of the *MS*50S and *MS*30S were refined against corresponding maps using real-space refinement (phenix.real_space_refine) (Afonine et al., 2012) in PHENIX (Adams et al., 2010). After the refinement, manual adjustments were conducted using COOT. These procedures were done in several rounds. Further refinement was carried out using REFMAC (Murshudov et al., 1997) in Fourier space according to the previously published protocols (Amunts et al., 2014). Cross validations were also performed following the same procedure described elsewhere (Wu et al., 2016). The final models were evaluated using MolProbity (Chen et al., 2010).

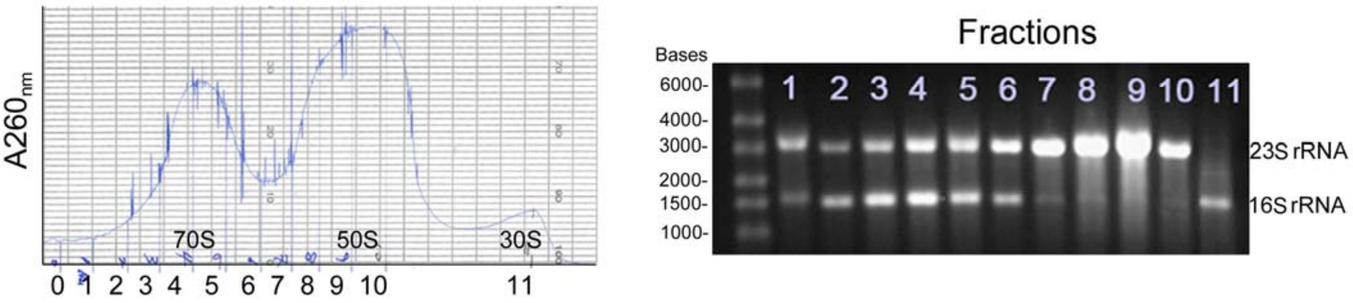

Figure S1. **Purification of *M. smegmatis* 70S ribosome.**

(A) The elution profiles of *M. smegmatis* ribosome after sucrose gradient (10% to 40% m/v) centrifugation in Beckman coulter Ti 15 rotor for 16 hours at 26,000 rpm. (B) The rRNA from individual fractions were isolated with 1:1 phenol/chloroform treatment and confirmed by 1% agarose gel. Both 16S rRNA and 23S rRNA were clearly visible representing 30S and 50S subunit respectively. 70S ribosome from the factions 1-6 was collected after pelleting by centrifugation in Beckman coulter Ti 50.2 rotor for 24 hours at 35,000 rpm.

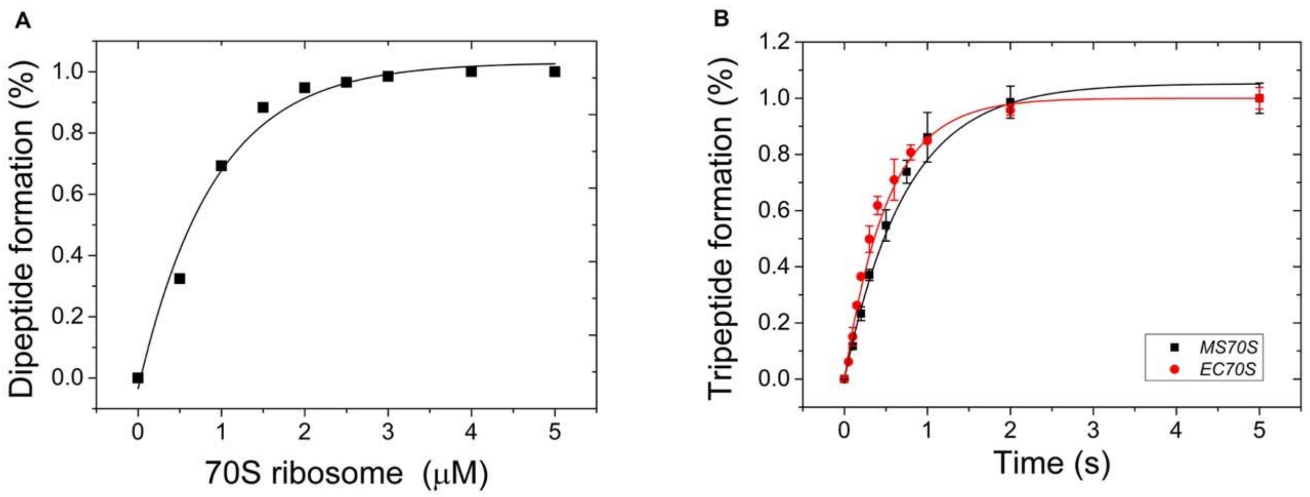

Figure S2. **Activity of purified *M. smegmatis* 70S ribosomes in dipeptide and tripeptide synthesis.**

(A) Single round dipeptide ([^{3}H]fMet-Leu) formation with *M. smegmatis* ribosome (*MS*70S) and translation factors. The active fraction of the ribosome was determined by the reciprocal of the minimal ribosome concentration at the saturation.

(B) Time course of EF-G dependent tripeptide ([^{3}H]fMet-Leu-Leu) formation starting from the initiation complex with ribosome and all translation factors from *M. smegmatis* (black box, *MS*70S) and *E. coli* (red circle, *EC*70S). The data are fitted with a single exponential leading to the rates $k_{obs} = 1.36 \pm 0.12$ s^{-1} for *MS*70S *and* $k_{obs} = 2.8 \pm 0.2$ s^{-1} for *EC*70S reactions.

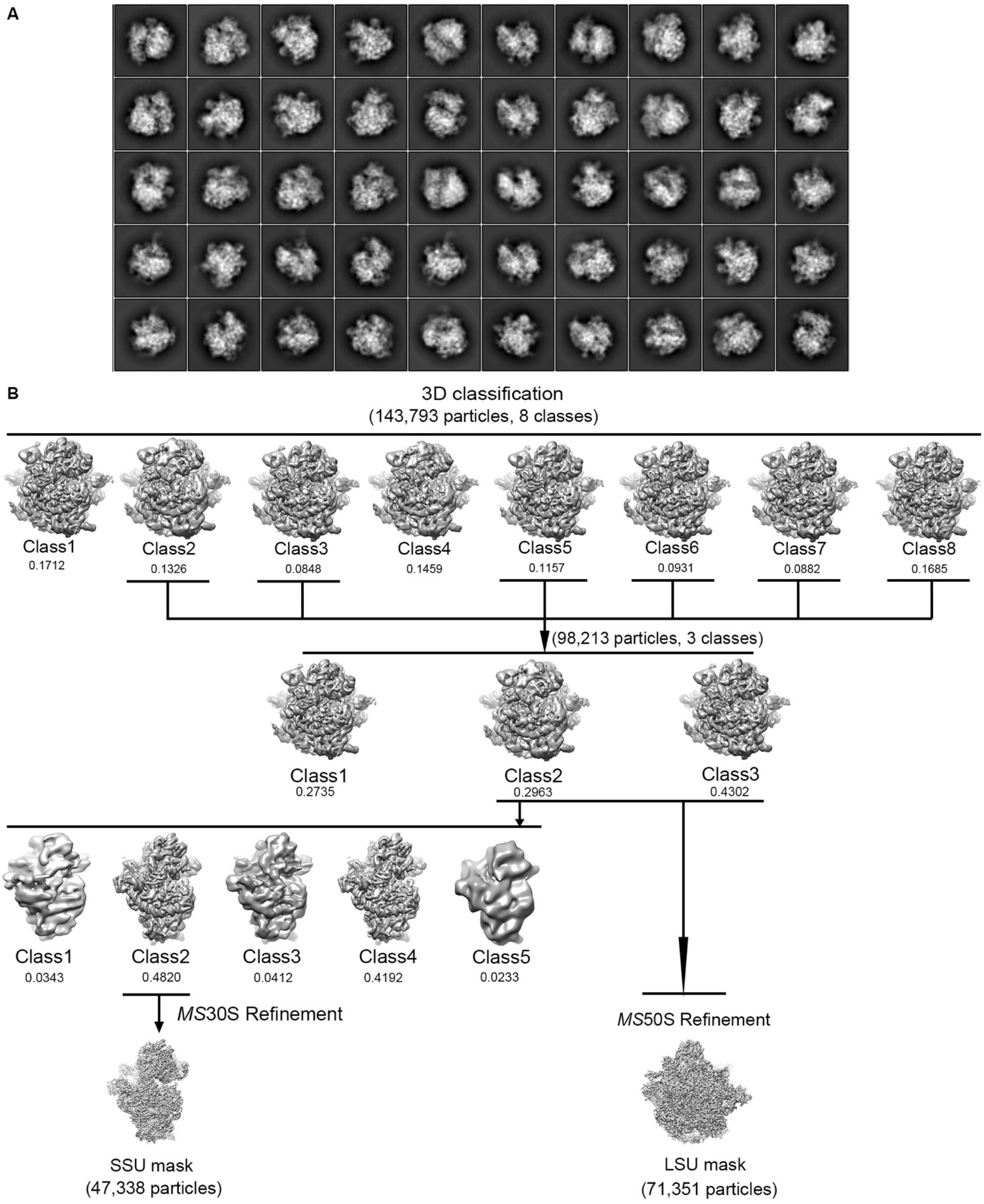

Figure S3. Cryo-EM data processing of the *M. smegmatis* ribosome particles.

(A) Representative 2D class averages of the *M. smegmatis* ribosome particles. (B) A flow chart for the 3D classification of the *M. smegmatis* ribosome particles (see methods for details).

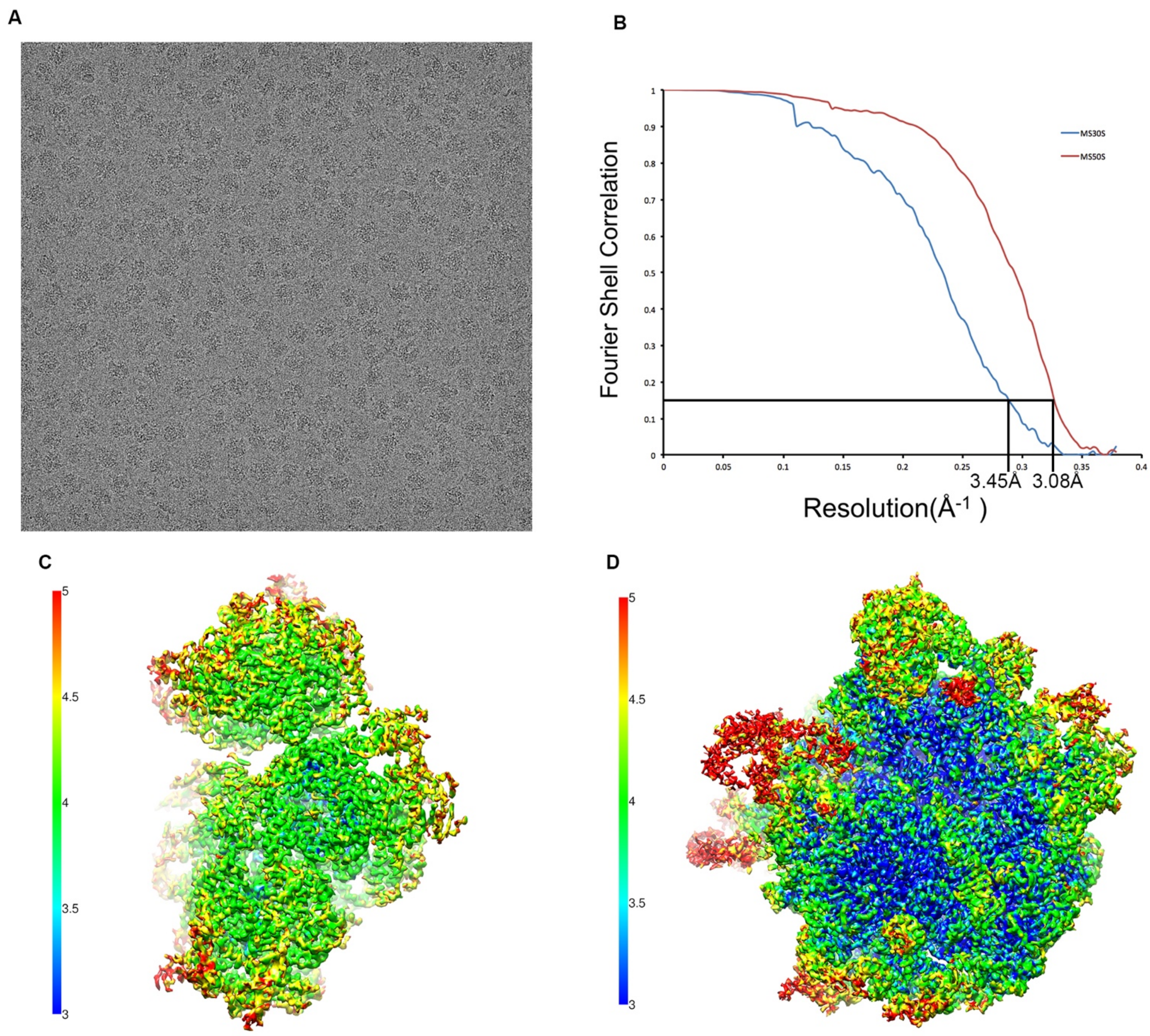

Figure S4. Resolution estimation of the final *M. smegmatis* ribosomal maps.

(A) A representative raw micrograph of *M. smegmatis* ribosome particles in vitreous ice. (B) Fourier Shell Correlation curves for the final density maps from mask-based refinement. (C, D) Local resolution maps of the *MS*30S (c) and *MS*50S (d) density map.

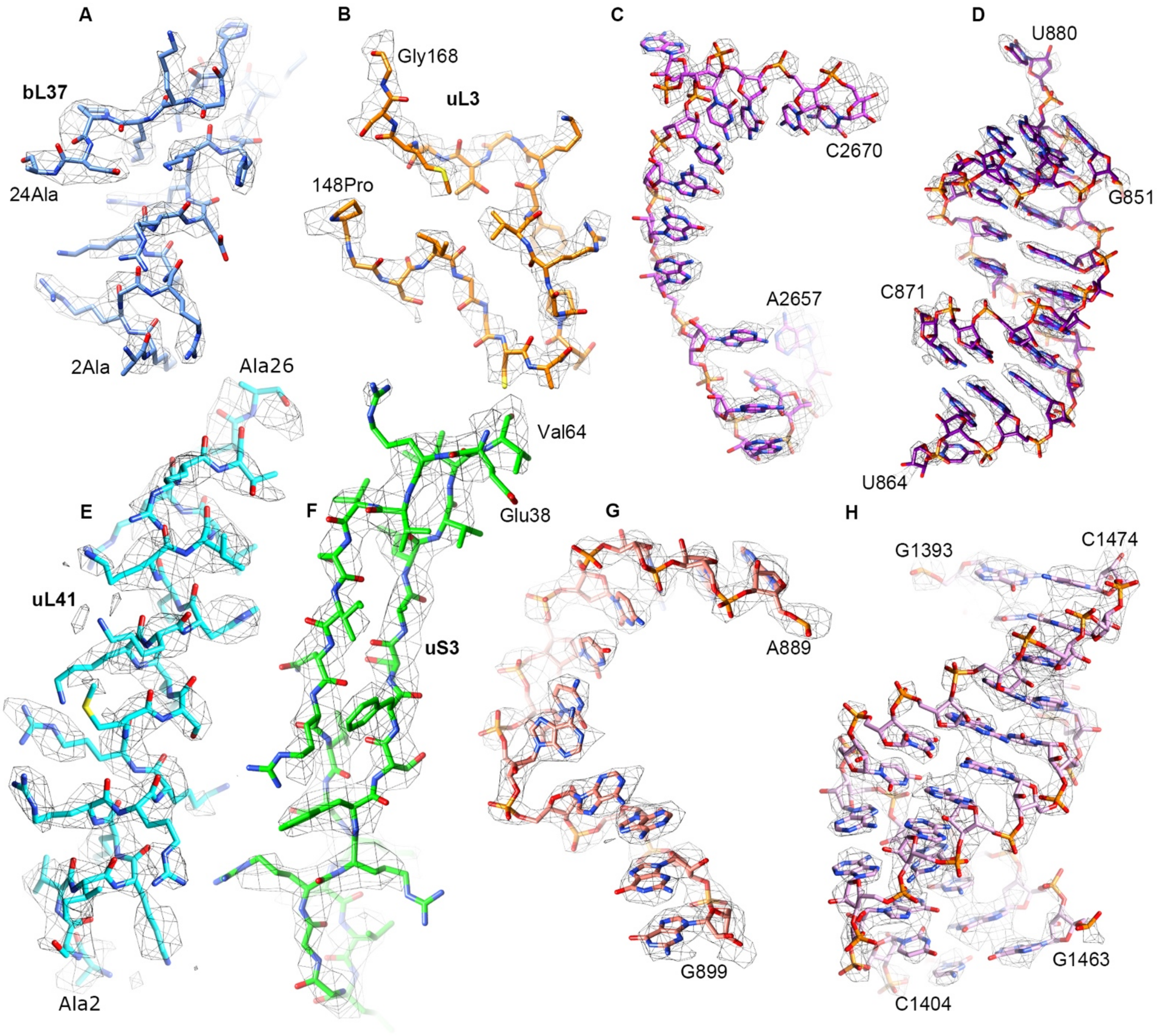

Figure S5. Representative densities of different elements in the density map of the *M. smegmatis* ribosome.

(A-D) Local densities of representative regions from the *MS*50S. (E-H) Local densities of representative regions from the *MS*30S. (A) The density of protein bL37. (E) The density of protein uL41.

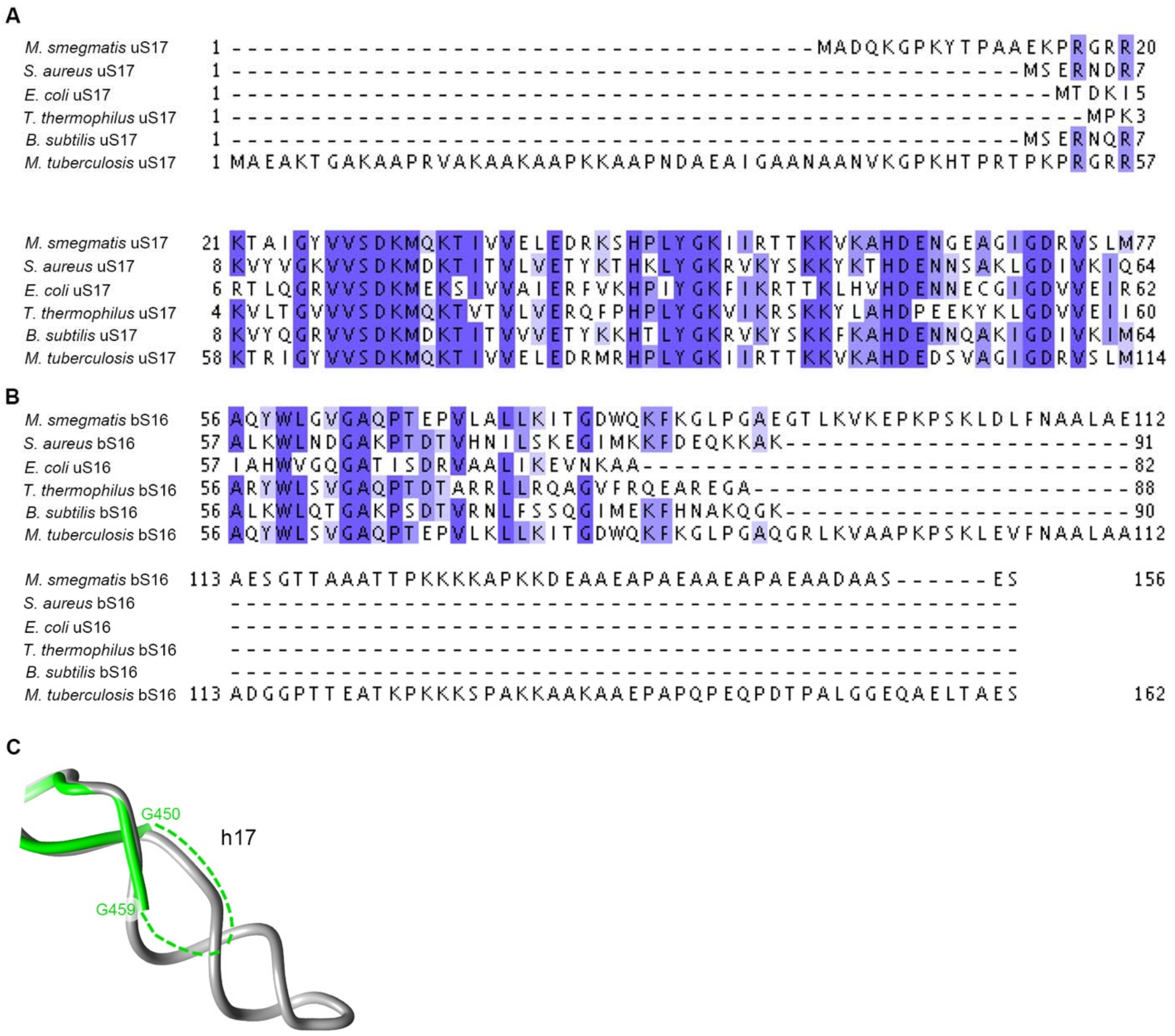

Figure S6. Cross-species comparison of representative proteins and rRNA helix in the *M. smegmatis* 30S.

(A, B) sequence alignment of uS17 and bS16 from different species. (C) Comparison of the shorter h17 in the *MS*30S (green) with its longer counterpart in the *EC*30S (gray).

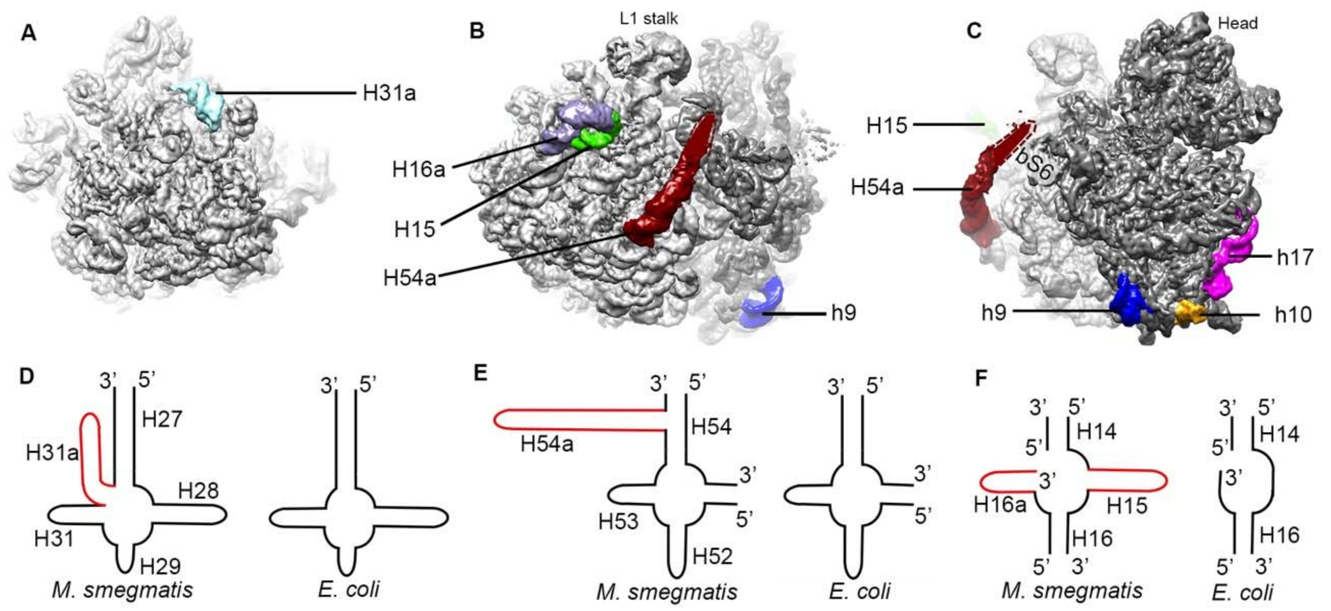

Figure S7: Mycobacterium-specific rRNA extensions in the *MS*70S.

(A-C) The low-pass filtered density map of the *MS*70S is displayed in surface representation (50S and 30S in light and dark grey, respectively), viewed from the 50S solvent surface (A), the *MS*70S L1-stalk region (B) and the *MS*30S solvent surface (C). Mycobacterium-specific rRNA extension segments are highlighted in different colors (H31a in cyan, H16a in purple, H15 in green, H54a in red, h9 in blue, h10 in yellow and h17 in pink).

(D-F) Comparison of mycobacterium-specific rRNA extension segments with their *E. coli* counterparts.

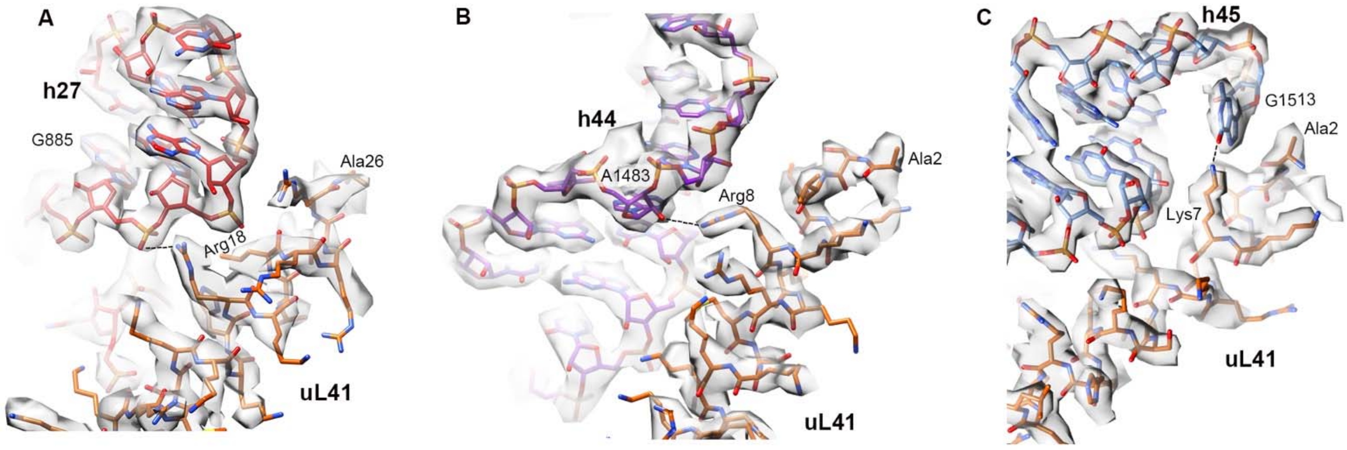

Figure S8. Specific contacts between uL41 and surrounding helices from the decoding center.

(A-C) Local interactions between uL41 and h27 (A), h44 (B), and h45 (C). A few selected interacting residues are labeled.

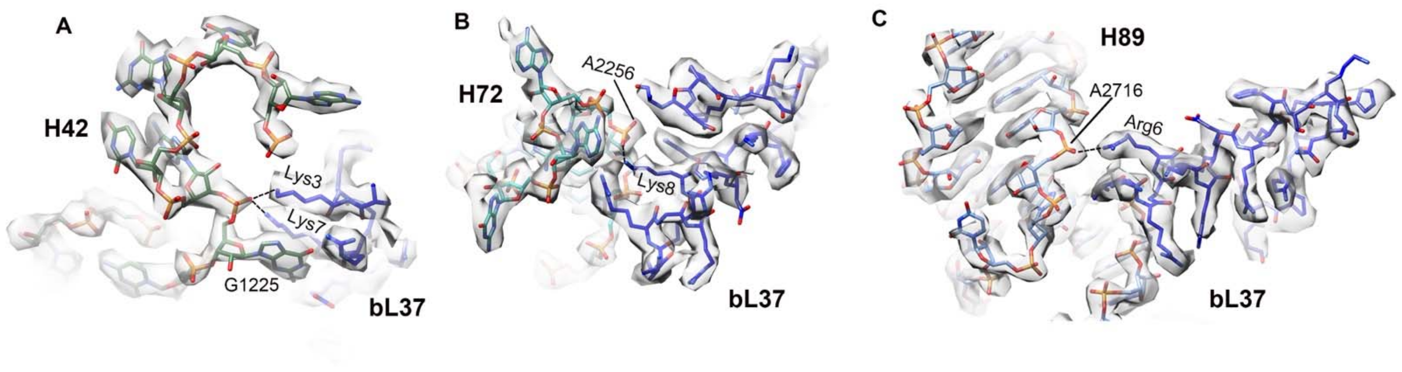

Figure S9. Specific contacts between bL37 and surrounding helices from the 50S subunit.

(A-C) Local interactions between uL37 and H42 (A), H72 (B), and H89 (C). A few selected interacting residues are labeled.

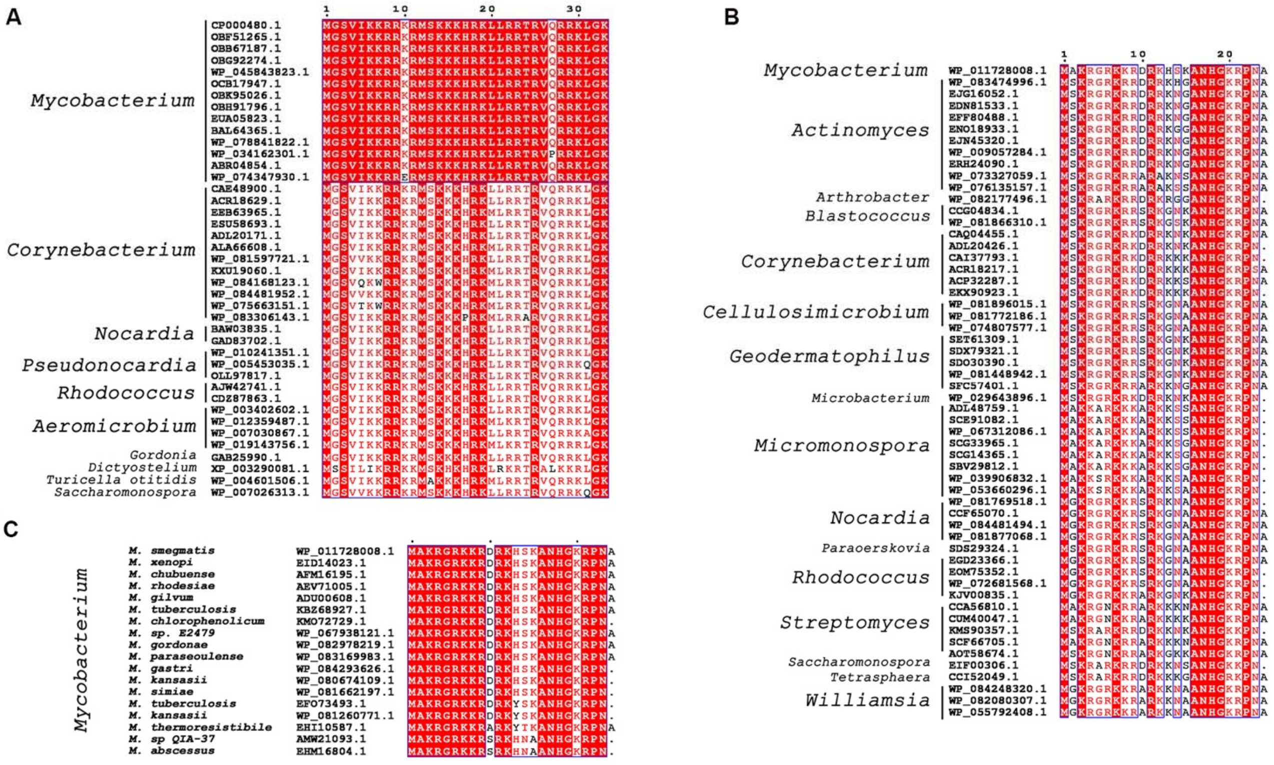

Figure S10. The distribution of uL41 (A) and bL37 (B and C) in bacteria based on NCBI non-redundant protein sequence homology searching. The sequence was aligned using EMBL-EBI Clustal Omega and input with ESPript 3 (Robert and Gouet, 2014). The identical (strictly conserved) and similar residues were boxed in red and white respectively. The classification of accession number for various bacteria is based on NCBI Taxonomy.

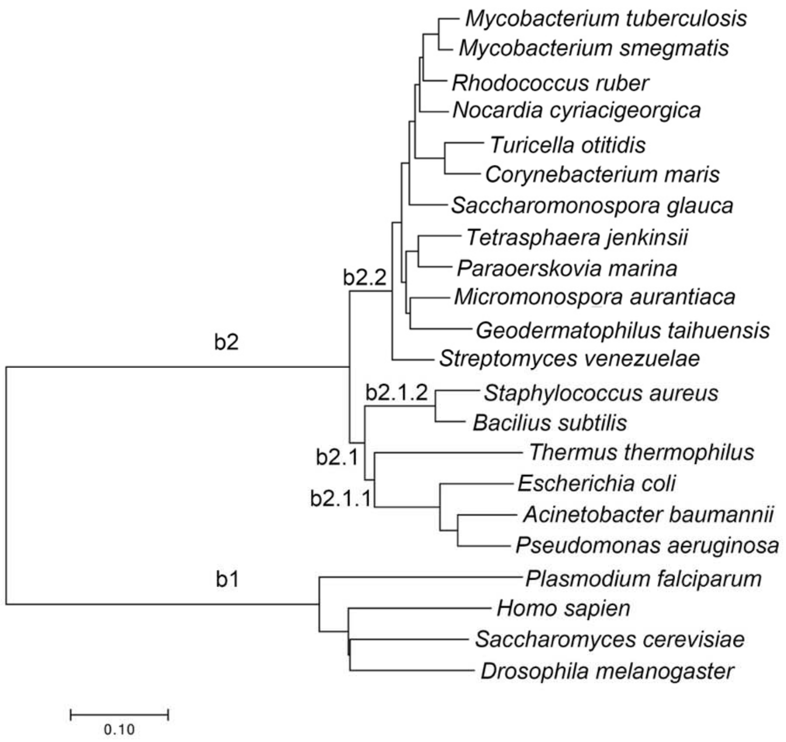

Figure S11. Evolution relationship based on 16S-like rRNA among bacteria and eukaryotes.

Branch b1 represents eukaryotes, and branch b2 represents bacteria. Branch b2.2 is actinobacteria and they have proteins uL41 and bL37. The evolution tree was generated using the software MEGA7 (Kumar et al., 2016).

Chain ID	Protein name	Uniprot ID OR Genebank ID	Total number of residues	Residue built	Extension compared to EC	Extension compared to SA	Extension compared to TT	Extension compared to BS	Extension compared to MT
C	uS3	ABK75343	275	2-42;51-74; 81-203	234-275	219-275	238-275	220-275	
D	uS4	ABK70484	201	2-18;46-146; 173-185;191-200					
E	uS5	ABK71824	214	35-193	1-25;193-214	1-25;192-214	1-30;193-214	1-25;192-214	
F	bS6	ABK72345	90	1-10;29-85					
G	uS7	ABK74236	156	2-79;86-153					
H	uS8	ABK75423	132	2-132					
I	uS9	ABK70660	150	25-149	1-20	1-18	1-22	1-20	
J	uS10	ABK75640	101	4-101					
K	uS11	ABK69610	138	22-138	1-7	1-9	1-9	1-7	
L	uS12	ABK73485	124	2-124					
M	uS13	ABK76170	124	2-113					
N	uS14	ABK73792	101	2-61		1-38	1-38	7-44	1-38
O	uS15	ABK73765	89	2-87					
P	bS16	ABK69801	156	2-96	82-156	89-155	89-156	91-156	
Q	uS17	ABK72361	98	5-97	1-15	1-17	1-17	1-13	
R	bS18	ABK72284	84	27-81	1-9				
S	uS19	ABK73644	93	4-82					
T	bS20	ABK69738	86	3-85					
U	uL41	ABK74558	33	2-26					

Table S1: Ribosomal proteins in the model of the *MS*30S.

Chain ID	Protein name	Uniprot ID OR Genebank ID	Total number of residues	Residue built	Extension compared to EC	Extension compared to SA	Extension compared to TT	Extension compared to BS	Extension compared to MT
C	uL2	ABK74446	278	2-274					
D	uL3	ABK75373	217	3-214			89-95		
E	uL4	ABK72271	215	2-209			24-28		
F	uL5	ABK75083	187	9-184	1-7	1-7			
G	uL6	ABK73224	179	13-26;31-38; 59-176					
H	bL9	ABK76149	151	1-9;15-39					
J	uL13	ABK73633	147	2-147					
K	uL14	ABK72408	122	1-122					
L	uL15	ABK72911	147	3-146					
M	uL16	ABK74245	138	1-135					
N	bL17	ABK73583	199	2-117	117-199	117-199	118-199	117-199	171-178;192-199
O	uL18	ABK74050	127	3-65;77-127	1-11	1-7	1-11		
P	bL19	ABK69938	113	2-112					
Q	bL20	ABK72978	129	2-125	119-129	119-129	119-129	120-129	
R	bL21	ABK75887	103	3-102					
S	uL22	ABK71298	153	7-117	1-7;118-153	1-7;125-153	1-7;121-153	1-7;121-153	
T	uL23	ABK70487	100	4-69;72-96					
U	uL24	ABK73139	105	1-43;61-88; 94-105					
V	bL25	ABK75240	215	7-183	1-7;101-215	1-7			
W	bL27	ABK73657	88	8-83					

X	bL28	A0QV03_MYCS2	64	2-63					
Y	uL29	ABK73490	77	6-66	68-77		70-77		
Z	uL30	ABK75416	61	2-59					
0	bL32	ABK71255	57	2-56					
1	bL33	ABK75620	54	7-18;21-54					
2	bL34	ABK71603	47	2-47					
3	bL35	ABK75420	64	2-64					
4	bL36	ABK75604	37	1-37					
a		ABK73049	24	2-24					

Table S2: Ribosomal proteins in the model of the *MS*50S.

Supplementary References

Adams, P.D., Afonine, P.V., Bunkoczi, G., Chen, V.B., Davis, I.W., Echols, N., Headd, J.J., Hung, L.W., Kapral, G.J., Grosse-Kunstleve, R.W.*, et al.* (2010). PHENIX: a comprehensive Python-based system for macromolecular structure solution. Acta Crystallogr D *66*, 213-221.

Afonine, P.V., Grosse-Kunstleve, R.W., Echols, N., Headd, J.J., Moriarty, N.W., Mustyakimov, M., Terwilliger, T.C., Urzhumtsev, A., Zwart, P.H., and Adams, P.D. (2012). Towards automated crystallographic structure refinement with phenix.refine. Acta Crystallogr D *68*, 352-367.

Amunts, A., Brown, A., Bai, X.C., Llacer, J.L., Hussain, T., Emsley, P., Long, F., Murshudov, G., Scheres, S.H.W., and Ramakrishnan, V. (2014). Structure of the Yeast Mitochondrial Large Ribosomal Subunit. Science *343*, 1485-1489.

Carter, A.P., Clemons, W.M., Brodersen, D.E., Morgan-Warren, R.J., Wimberly, B.T., and Ramakrishnan, V. (2000). Functional insights from the structure of the 30S ribosomal subunit and its interactions with antibiotics. Nature *407*, 340-348.

Chen, V.B., Arendall, W.B., 3rd, Headd, J.J., Keedy, D.A., Immormino, R.M., Kapral, G.J., Murray, L.W., Richardson, J.S., and Richardson, D.C. (2010). MolProbity: all-atom structure validation for macromolecular crystallography. Acta Crystallogr D Biol Crystallogr *66*, 12-21.

Degiacomi, G., Personne, Y., Mondesert, G., Ge, X., Mandava, C.S., Hartkoorn, R.C., Boldrin, F., Goel, P., Peisker, K., Benjak, A.*, et al.* (2016). Micrococcin P1 - A bactericidal thiopeptide active against Mycobacterium tuberculosis. Tuberculosis (Edinb) *100*, 95-101.

Emsley, P., Lohkamp, B., Scott, W.G., and Cowtan, K. (2010). Features and development of Coot. Acta Crystallogr D Biol Crystallogr *66*, 486-501.

Holm, M., Borg, A., Ehrenberg, M., and Sanyal, S. (2016). Molecular mechanism of viomycin inhibition of peptide elongation in bacteria. Proc Natl Acad Sci U S A *113*, 978-983.

Kucukelbir, A., Sigworth, F.J., and Tagare, H.D. (2014). Quantifying the local resolution of cryo-EMEM density maps. Nature Methods *11*, 63-+.

Kumar, S., Stecher, G., and Tamura, K. (2016). MEGA7: Molecular Evolutionary Genetics Analysis Version 7.0 for Bigger Datasets. Mol Biol Evol *33*, 1870-1874.

Li, X., Mooney, P., Zheng, S., Booth, C.R., Braunfeld, M.B., Gubbens, S., Agard, D.A., and Cheng, Y. (2013). Electron counting and beam-induced motion correction enable near-atomic-resolution single-particle cryo-EM. Nat Methods *10*, 584-590.

Li, X., Zheng, S., Agard, D.A., and Cheng, Y. (2015). Asynchronous data acquisition and on-the-fly analysis of dose fractionated cryoEM images by UCSFImage. J Struct Biol *192*, 174-178.

Mandava, C.S., Peisker, K., Ederth, J., Kumar, R., Ge, X., Szaflarski, W., and Sanyal, S. (2012). Bacterial ribosome requires multiple L12 dimers for efficient initiation and elongation of protein synthesis involving IF2 and EF-G. Nucleic Acids Res *40*, 2054-2064.

Mindell, J.A., and Grigorieff, N. (2003). Accurate determination of local defocus and specimen tilt in electron microscopy. J Struct Biol *142*, 334-347.

Murshudov, G.N., Vagin, A.A., and Dodson, E.J. (1997). Refinement of macromolecular structures by the maximum-likelihood method. Acta Crystallogr D *53*, 240-255.

Pulk, A., and Cate, J.H. (2013). Control of ribosomal subunit rotation by elongation factor G. Science *340*, 1235970.
Robert, X., and Gouet, P. (2014). Deciphering key features in protein structures with the new ENDscript server. Nucleic Acids Research *42*, W320-324.
Scheres, S.H. (2012). RELION: implementation of a Bayesian approach to cryo-EM structure determination. J Struct Biol *180*, 519-530.
Shaikh, T.R., Gao, H., Baxter, W.T., Asturias, F.J., Boisset, N., Leith, A., and Frank, J. (2008). SPIDER image processing for single-particle reconstruction of biological macromolecules from electron micrographs. Nat Protoc *3*, 1941-1974.
Sievers, F., Wilm, A., Dineen, D., Gibson, T.J., Karplus, K., Li, W., Lopez, R., McWilliam, H., Remmert, M., Soding, J.*, et al.* (2011). Fast, scalable generation of high-quality protein multiple sequence alignments using Clustal Omega. Mol Syst Biol *7*, 539.
Wu, S., Tutuncuoglu, B., Yan, K., Brown, H., Zhang, Y., Tan, D., Gamalinda, M., Yuan, Y., Li, Z., Jakovljevic, J.*, et al.* (2016). Diverse roles of assembly factors revealed by structures of late nuclear pre-60S ribosomes. Nature *534*, 133-137.
